# Acoustic Speech Analysis in Alzheimer’s Disease: A Systematic Review and Meta-Analysis

**DOI:** 10.14283/jpad.2024.132

**Published:** 2024-08-13

**Authors:** S. Saeedi, S. Hetjens, M. O. W. Grimm, Ben Barsties v. Latoszek

**Affiliations:** 1 Independent Researcher in Laryngology, Voice Pathology, and Speech-Language Pathology, Tehran, Iran; 2 Department for Medical Statistics and Biomathematics, Medical Faculty Mannheim, University of Heidelberg, Mannheim, Germany; 3 Experimental Neurology, Saarland University, 66424 Homburg, Germany; 4 Nutrition Therapy and Counseling, SRH University of Applied Health Sciences, Campus Rheinland, 51377 Leverkusen, Germany; 5 Speech-Language Pathology, SRH University of Applied Health Sciences, Düsseldorf, Germany; 6 Graf-Adolf-Straße 67, 40210 Düsseldorf, Germany

**Keywords:** Alzheimer’s disease, diagnosis, acoustic, signal processing, speech and voice analysis

## Abstract

**Background:**

The potential of biomarkers in the detection of Alzheimer’s disease (AD) is prominent. Acoustics may be useful in this context but the evaluation and weighting for specific acoustic parameters on continuous speech is missing. This meta-analysis aimed to explore the significance of acoustic parameters from acoustic speech analysis on continuous speech, as a diagnostic tool for clinical AD.

**Methods:**

Applying PRISMA protocol, a comprehensive search was done in MEDLINE, Scopus, Web of Science, and CENTRAL, from 1960 to January 2024. Cross-sectional studies comparing the acoustic speech analysis between AD patients and healthy controls (HC), were taken into account. The bias risk of the included studies were examined via JBI checklist. Using Review Manager v.5.4.1, the mean differences of acoustic speech parameters among AD and HC were weighted, and the pooled analysis and the heterogeneity statistics were conducted.

**Results:**

In total, 1112 records (without duplicates) were obtained, and 11 papers with 7 acoustic parameters were included for this study, and 8 from 11 studies were identified with a low level of bias. Five from 7 acoustic parameters revealed significant differences among the two groups (p-values ≤ 0.01), in which for all rate-related and interruption-related acoustic parameters were the most prominent and less in temporal-related acoustic parameters.

**Conclusions:**

Although a small number of acoustic parameters on continuous speech could be evaluated in the detection of clinical AD, the greatest potential of acoustic biomarkers for AD appeared to exist in two of three categories. Further contributions of high-quality studies are needed to support evidence for acoustics as biomarkers for AD.

**Electronic Supplementary Material:**

Supplementary material is available in the online version of this article at 10.14283/jpad.2024.132.

## Introduction

Alzheimer’s disease (AD) is a progressive neurodegenerative disorder that significantly impacts cognitive function, leading to severe impairment in daily living activities. It is the most common cause of dementia among older adults, characterized by a gradual decline in memory, language, problem-solving abilities, and other critical thinking skills. The exact cause of AD remains largely unknown, although it is believed to result from a combination of genetic, environmental, and lifestyle factors. The early detection of AD poses a significant challenge yet is crucial for managing symptoms and planning care.

Over the last few decades, significant progressions have been achieved in the field of biomarker development regarding AD diagnosis. Prominent examples include the identification of biomarkers such as analyzing proteins, the utilization of neuroimaging techniques, and the implementation of neuropsychological tests (1–3). Beside neurofilament light chain, both the amyloid-b (Ab) peptide as well as hyperphosphorylated forms of the microtubule-associated protein tau play an important role as biomarkers, as senile plaques consisting of aggregated Ab-peptides and neurofibrillary tangles which consist of hyperphosphorylated tau proteins represent the main histopathological hallmarks of AD (4, 5). Notably, these proteins can be detected in cerebrospinal fluid as well as blood of AD patients and can be detected in vivo by PET (positron emission tomography) scans (6, 7). Nevertheless, these diagnostic methods are constrained by their substantial financial burden on the healthcare systems of countries and also invasive characteristics (8). Therefore, AD is often diagnosed based on clinical criteria, such as the National Institute on Aging-Alzheimer’s Association (NIA-AA) criteria, which rely on the presence of characteristic cognitive and functional impairments. Thus, it is important to note that there is a distinction between the biological and clinical diagnoses of AD. While the biological diagnosis is based on the presence of AD neuropathology, as evidenced by biomarkers, the clinical diagnosis is based on the presence of characteristic symptoms and signs of the disease. Some studies have shown that a significant proportion of individuals with a clinical diagnosis of AD may not have evidence of AD neuropathology on biomarker testing (9, 10). Conversely, some individuals with biomarker evidence of AD neuropathology may not meet clinical criteria for AD due to the absence of significant cognitive or functional impairments.

Due to the noted speech and language impairments observed in the initial phases of AD (11, 12), a question arises as to whether or not acoustic speech analysis can be used to assess and screen for AD. In recent years, the importance of speech signal processing in medical practice has gained considerable attention, as indicated by the numerous studies conducted on the topic (13). Advancements in technology and the availability of relevant user-friendly software packages have led to an increase in the clinical use of these measurements (14). Speech signal processing can provide valuable information regarding different aspects of speech signal such as speech rate, pitch and loudness variability, quality, resonance, articulation precision, and regularity (15–17). Acoustic measurements can be performed with easily available recording devices, making it a cost-effective and easily accessible tool. In addition, acoustics offers an objective analysis of quantitative measurements that have great potential for future developments in the field of diagnostics research.

The purpose of this meta-analysis is to assess acoustic speech parameters on continuous speech material in the detection of AD. The aim of the study was to comprehensively analyze the existing literature on this topic and identify the strengths and limitations of current research. By synthesizing the available evidence, this paper aimed to provide a comprehensive understanding of the potential benefits of speech signal processing in the identification of AD. Furthermore, this study seeks to bridge the gap between traditional biomarker research and the emerging field of acoustic analysis, highlighting the potential for non-invasive, cost-effective screening methods. The integration of acoustic biomarkers could significantly enhance early detection strategies, ultimately contributing to improved patient care and management. The exploration of acoustic parameters not only enriches our diagnostic toolbox but also underscores the multidisciplinary approach necessary for tackling complex diseases like AD. Through meticulous analysis and evaluation, this meta-analysis endeavors to elevate the discourse surrounding acoustic biomarkers and lay the groundwork for future investigations that could redefine diagnostic paradigms for AD.

## Materials and Methods

### Data sources and searches

The conduction of this review followed the guidelines outlined in the Preferred Reporting Items for Systematic Reviews and Meta-Analyses (PRISMA) statement (18). Relevant studies were identified by a systematic search electronic databases from MEDLINE (PubMed), Scopus, Web of Science, and Cochrane Central Register of Controlled Trials (CENTRAL), starting from 1960 until January 2024. Different key words were combined relating to acoustics measurements of speech analysis on continuous speech between Alzheimer’s disease and healthy controls (HC). Appendix 1 contains a comprehensive list of selected keywords with Boolean operators used in each database.

We identified articles through the review of their titles and abstracts. A systematic search was then undertaken for scientific reports published in English, and only those located within the databases were incorporated into the meta-analysis.

### Study Selection

This study comprised cross-sectional investigations that examined differences in the outcomes of acoustic speech measures on continuous speech material between AD and HC. The features of the quantitative acoustic measures for inclusion represent gender-independence and provide information on prosodic (alterations in rhythm, timing, stress, and pitch) and quality-related aspects of speech. Moreover, it is important to acknowledge that the included studies might rely mostly on clinical diagnoses of AD, and biomarker confirmation of the underlying AD neuropathology would be low. As such, the results of this meta-analysis should be interpreted as distinguishing between individuals with and without dementia, rather than specifically between those with and without biological AD.

Studies were excluded if: [1] a study group of participants with mild cognitive impairment or other types of dementia that are not associated with or do not present with the (clinical) symptoms of Alzheimer’s disease; [2] published studies were written in a language other than English; [3] studies that were not published in a peer-reviewed journal; [4] studies were either traditional narrative, or systematic reviews, case studies, or contained other study designs than cross-sectional studies.

### Risk of Bias Assessment

The evaluation of bias in the studies included was conducted using a checklist for cross-sectional studies provided by the Joanna Briggs Institute (JBI) Critical Appraisal Checklist for Analytical Cross-Sectional Studies which consists of 8 items (19). The checklist items assess the study sample (inclusion criteria, detailed description of subjects, and the study setting), exposure and outcomes measures (validity and reliability of measuring exposure and outcome, and criteria used for measurement), confounding bias (identification of confounding factors, and strategies to deal with them), and data analysis (appropriateness of statistical analysis). The possible responses for each 8 items are «yes,» «unclear,» «no,» or «not applicable.» Before starting the critical appraisal, all reviewers agreed on the scoring decisions based on the previous studies (19, 20). The studies were then grouped according to the following criteria: (a) studies with a score of above 70% «yes» were considered to have a low risk of bias, (b) studies with «yes» scores between 50% and 69% were categorized as having a moderate risk of bias, and (c) studies with «yes» scores below 49% were determined to have a high risk of bias.

### Data Extraction

Two reviewers (SS and BBvL) were responsible for extracting the data. Both reviewers independently evaluated the titles and abstracts of the retrieved studies. In the subsequent screening phase, a comprehensive analysis and evaluative assessment were conducted on the full text of the chosen studies. This meticulous process aims to ascertain the suitability of including these studies in the meta-analysis. The collected information from the chosen studies encompassed various aspects, including article attributes (such as authors, publication year, journal, and paper title), study characteristics (such as research design, sample size, participants with AD in comparison with cognitively normal individuals, speech task, software, and acoustic data), patient demographics (age and gender), and outcomes of the various acoustic measures of speech analyses. Any disagreements between the reviewers were resolved through discussion. Articles that did not meet the inclusion criteria were excluded.

### Statistics

The program Review Manager (RevMan), version 5.4.1 (The Nordic Cochrane Centre, The Cochrane Collaboration, Copenhagen, 2020) was used for the statistical analyses of the meta-analysis on the final selected results of acoustic measures from the systematic search listed in Table 1, which were assessed more than once. First, the difference between HC and AD was calculated of the outcomes of the specific acoustic measures. This difference was weighted according to the DerSimonian & Laird method (21). Second, the results of the pooled analysis and the heterogeneity statistics were presented in a forest plot. The heterogeneity of studies was calculated using the I^2^ index. An I^2^ value of 0 – 25 % represents insignificant heterogeneity; > 25 % – 50 % low heterogeneity; > 50 % – 75 % moderate heterogeneity; and > 75 % high heterogeneity (22). Analyses with insignificant heterogeneity were calculated using a fixed-effects model, otherwise with random effects model. A p-value of less than 0.05 was considered as statistically significant.

**Table 1:** Characteristics of cross-sectional trials in the meta-analysis

**Source**	**Sample Size**	**Cognition Status**	**Age (Mean/Range in Years)/Gender**	**Speech Task & Language**	**Software**	**Parameters**
Meilán et al. (2014) (23)	Total: n = 66	AD (n = 30) diagnosis with NINCDS-ADRDA criteria; HC (n = 36)	AD (78.66/60–95); HC (74.06/60–98) F/M: 2.92	continuous speech (reading) Spanish	Praat	total duration; phonation time; speech rate; articulation rate; mean F0; minimum F0; maximum F0; autocorrelation; high global pitch; low global pitch; pulses periods; mean periods; without voice; voice breaks; voice breaks; proportion of pauses of voice; pauses of voice; jitter (loc); jitter (loc, abs); jitter (rap); jitter (ppq5); shimmer (loc); shimmer (apq3); shimmer (apq5); shimmer (apq11); intensity of unvoiced; intensity of voiced; noise-to-harmonics ratio; harmonics-to-noise ratio (HNR)
Gonzalez-Moreira et al. (2015) (24)	Total: n = 20	MD (n = 10) diagnosis with MMSE; HC (n = 10)	MD (80.3/N/A; HC (78.9/N/A) F/M: 0/33	continuous speech (reading) Spanish	DCGrab	speech time; number of pauses; proportion of pause; phonation time; proportion of phonation; speech rate; articulation rate; number of syllables; mean of syllables duration; SD of F0; maximum variation of F0; mean of F0
Martinez-Sanchez et al. (2017) (25)	Total: n = 127	AD (n = 45) diagnosis with NINCDS-ADRDA criteria; HC (n = 82)	AD (77.80/N/A; HC (75.83/N/A) F/M: 1.97	continuous speech (reading) Spanish	Praat	d average (average duration of syllabic intervals); ΔS (SD of syllabic intervals duration); Varco S (coefficient of variation of syllabic intervals duration); rPVI (coefficient of variation of syllabic intervals duration); Normalized Pairwise Variability Index (nPVI); d average (average duration of syllabic intervals); ΔS (SD of syllabic intervals
Martinez-Sanchez et al. (2018) (26)	Total: n = 145	AD (n = 47) diagnosis with NINCDS-ADRDA criteria; HC (n = 98)	AD (80.83/N/A; HC (76.18/N/A) F/M: 2.29	continuous speech (reading) Spanish	Praat	Acoustic Voice Quality Index (AVQI), AVQI NHR; F0 rank; amplitude mean; amplitude maximum difference mean; amplitude minimum; asymmetry; noise harmonic ratio; harmonic noise ratio SD; F1; F1 Hz SD; F3; F3 B3; TrajIntraZ; nPVI
De Loozea et al. (2018) (27)	Total: n = 54	AD (n = 18) diagnosis with NIA-AA criteria; HC (n = 36)	AD (72.38 /N/A; HC (71.13/N/A) F/M: 0.94	continuous speech (reading) English	Praat	sentence duration; articulation rate; speech rate; duration of within-sentence pauses; duration of pauses between pairs of sentences; mean speech segment duration; number of speech chunk; number of pauses; number of dysfluences
Meilán el al. (2018) (28)	Total: n = 144	AD (n = 42) diagnosis with NINCDS-ADRDA criteria; HC (n = 102)	AD (79.10/N/A; HC (75.54/N/A) F/M: 2.38	continuous speech (reading) Spanish	Praat	percentage of unvoiced segments (without harmonic nature) in voice signal; shimmer Apq11; shimmer Apq3; percentage of voice Breaks; F3 SD; HNR; autocorrelation
Qiao et al. (2020) (29)	Total: n = 44	AD (n = 20) diagnosis with NINCDS-ADRDA criteria; HC (n = 24)	AD (75.0/N/A; HC (71.4/N/A) F/M: 2.21	continuous speech (spontaneous) Mandarin Chinese	ASR software for cognitive impairment	total silence duration; total speech duration; percentage of silence duration; average duration of speech segments; maximum duration of speech segments; minimum duration of speech segments; average duration of phrasal segments; maximum duration of phrasal segments; minimum duration of phrasal segments; average duration of silence segments; maximum duration of silence segments; minimum duration of silence segments; average duration of hesitations; number of speech segments; number of phrasal segments; number of silence segments; number of hesitations; number of long pauses; number of short pauses; ratio of silence/speech counts; ratio of hesitation/speech counts; ratio of long pause/speech counts; ratio of short pause/speech counts; ratio of hesitation/phrasal counts
Bose et al. (2021) (30)	Total: n = 14	AD (n = 6) diagnosis with NINCDS-ADRDA criteria; HC (n = 8)	AD (66.5/N/A; HC (71.7/N/A) F/M: N/A	continuous speech (spontaneous) Bengali	Quantitative Production Analysis (QPA)	duration of the narrative; total number of words; words per minute; proportion of words in sentences; mean sentence length; proportion well-formed sentence; Embedding Index; number of narrative words, number of open class words; number of closed class words; proportion of open class words; proportion of closed class words; proportion of noun; proportion of noun; noun–verb ratio; proportion of noun; proportion of pronoun; proportion of pronoun to noun; proportion of verb; proportion of verb; proportion of non-finite verb; proportion of matrix verb; proportion of compound verb; proportion of adjective; proportion of adverb; proportion of postposition; number of reduplication; Noun Inflection Index; proportion of inflected noun; proportion of noun with one inflection; proportion of noun with two or more inflections; rate of definiteness marker; rate of case markers; proportion of definiteness marker; proportion of case markings; Verb Inflection Index; inflection score; inflection complexity score; word count; number of Correct Information Unit analyses; idea density; idea efficiency; repetitions; revisions; reformulations; total count of disruption of spontaneity
Frankenberg et al. (2021) (31)	Total: n = 246	AD (n = 99) diagnosis with NINCDS-ADRDA criteria; HC (n =147)	AD (66.5/N/A); HC (71.7/N/A) F/M: 0.86	continuous speech (spontaneous) German	N/A	mean speech segment duration; mean speech duration; silence to word ratio; word rate; phoneme rate; Brunet’s-Index; Honore’s Statistic
Cho et al. (2022) (32)	Total: n = 72	AD (n =44) diagnosis with NIA-AA criteria; HC (n = 28)	AD (62.0/N/A); HC (65.9 /N/A) F/M: 0.86	continuous speech (spontaneous) English	Praat	mean speech segment duration, mean pause segment duration, percent of speech; pause rate per minute; articulation rate; pitch range
Yamada et al. (2023) (33)	Total: n = 68	AD (n = 25) diagnosis with NIA-AA criteria; HC (n =43)	AD (75.40/N/A); HC (72.16/N/A) F/M: 0.86	continuous speech (spontaneous) Japanese	N/A	pause duration; phoneme rate; pause duration; pitch variation; variance of MFCC6; variance of MFCC11; variance of MFCC3

Abbreviations: n, number; AD, Alzheimer’s disease; NINCDS-ADRDA, National Institute of Neurological and Communicative Disorders and Stroke and the Alzheimer’s Disease and Related Disorders Association; HC, healthy controls; F, female; M, male; MMSE, Mini-Mental State Examination; MD, mild dementia; N/A, not applicable; SD, standard deviation; NIA-AA, National Institute on Aging and Alzheimer’s Association; MFCC, mel frequency cepstrum coefficient.

## Results

### Study Characteristics

**Figure 1:**
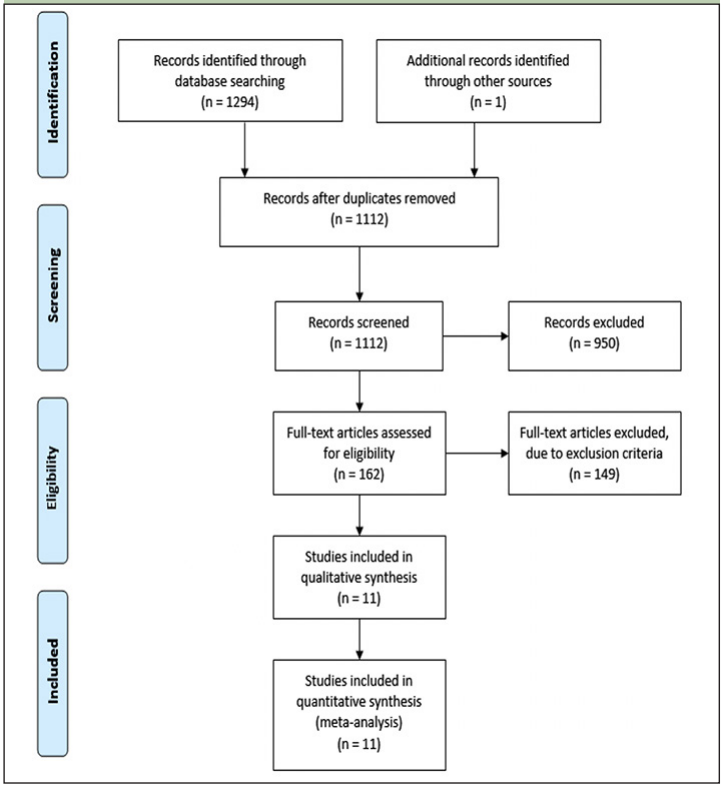
PRISMA flowchart of the process followed to select studies for the review

The PRISMA flowchart in Figure 1 provides a concise overview of the systematic search process. A comprehensive search yielded a total of 1,294 studies, of which 182 duplicates were identified and subsequently removed. After a thorough screening based on title and abstract, 162 studies were deemed suitable for a full-text review. However, after applying the specified exclusion criteria, 149 studies were excluded, resulting in 11 articles being included (see Table 1). Each study included a control group of healthy individuals and at least one experimental group of patients with AD. The acoustic measurements that were shared among the included studies provide quantitative information about various aspects of speech signals. For the present meta-analysis just 7 acoustic parameters could be analyzed from Table 1 as these parameters have been evaluated more than once in the same study context. These 7 acoustic parameters can be categorized into three groups: time (mean speech segment duration, mean pause duration, total duration, and normalized pairwise variability index (nPVI)), rate (speech rate, and articulation rate), and interruption (voice breaks) parameters (see Table 2).

**Table 2:** Details of acoustic parameters trials in the meta-analysis

**Categories**	**Acoustic Parameters**	**Description**
Temporal-related parameters	Mean speech segment duration	Average segment duration of continuous speech, excluding any pauses
	Mean pause duration	Average length of all pause segment durations presented in the speech
	Total duration	Overall length of time in the sample, which includes both periods of speech and pauses
	normalized pairwise variability index (nPVI)	This index is a measure which based upon the temporal and durational aspects of voice analyzing the normalized sequential variability of syllabic intervals, indicating a direct relationship with the level of rhythmic variability in speech
Rate-related parameters	Speech rate	Rate of speaking by measuring words per minute
	Articulation rate	Rate of articulation of each phoneme in a word with precision and clarity, excluding any pauses
Interruption-related parameter	Voice breaks	Measuring sudden momentary stops in producing the voice sound (instability) in percentage

In total 1000 voice samples of participants were evaluated, in which 386 volunteers were examined in studies involving AD and 614 healthy controls. From the 386 participants of the experimental group 289 had an AD diagnosis based NINCDS-ADRDA criteria, 87 with NIA-AA criteria, and 10 with clinical presentations based on clinical ratings such as Mini Mental State Examination. The number of participants ranging from 8 to 147 for HC and 6 to 99 for AD. The bias risk assessment is presented in Appendix 2 in which a majority of the included studies demonstrated a low risk of bias (n=8), and rest of them showed a moderate risk of bias (n=3).

### Meta-analysis

**Figure 2:**
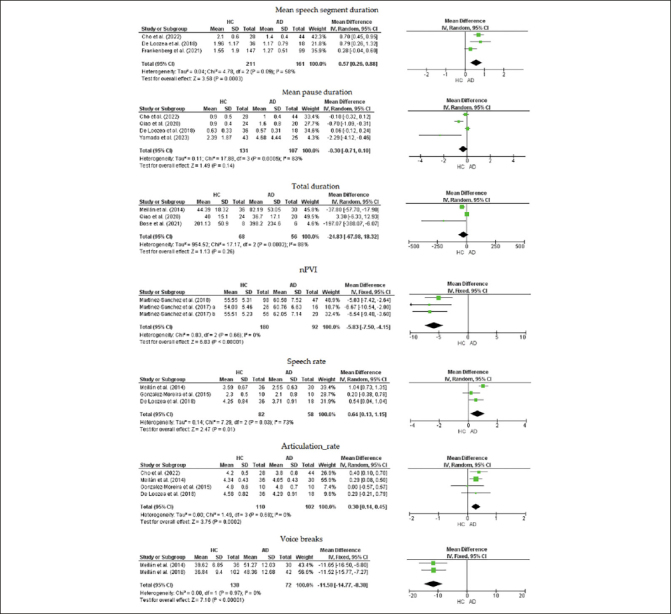
Forest plots of speech signal measurements Abbreviations: Martinez-Sanchez et al. (2017) a, only male participants of the study by Martinez-Sanchez et al. (2017); Martinez-Sanchez et al. (2017) b, only female participants of the study by Martinez-Sanchez et al. (2017)

Figure 2 shows the results for the seven acoustic parameters: Mean speech segment duration, mean pause duration, speech rate, articulation rate, total duration, nPVI and voice breaks. The mean difference (MD) of nPVI between healthy controls and AD was significantly slower in the healthy controls: MD = −5.83 (95% CI: −7.50 to −4.15, p < 0.0001). The voice breaks was significantly less in healthy controls than in AD: MD = −11.58 % (95% CI: −14.77 % to, −8.38 %, p < 0.0001). The following acoustic parameters were significantly faster in healthy controls than in AD: articulation rate: MD = 0.30 (95% CI: 0.14 to 0.45, p = 0.0002), mean speech segment duration: MD = 0.57 seconds (95% CI: 0.26 seconds to 0.88 seconds, p = 0.0003), and speech rate: MD = 0.64 (95% CI: 0.13 to 1.15, p = 0.0100). Mean pause duration and total duration were not significantly different between the two groups: MD = −0.30 seconds (95% CI: −0.71 seconds to 0.10 seconds, p = 0.1400), and MD = −24.83 seconds (95% CI: −67.98 seconds to 18.32 seconds, p = 0.2600), respectively. No heterogeneity was present in articulation rate, nPVI, and voice breaks. However, there was moderate heterogeneity in mean speech segment duration and high heterogeneity in mean pause duration, speech rate, and total duration.

## Discussion

The present study assessed the detection of Alzheimer’s disease in comparison to healthy controls by summarizing statistically the outcomes of seven speech acoustic parameters of the categories time, rate, and interruption. The neurodegenerative disease Alzheimer’s primarily affects memory and cognitive functions, but also various regions and centers in the brain that are responsible for language deficits (e.g. naming and word retrieval) such as bilateral inferior frontal gyrus (IFG), right superior frontal gyrus (SFG), the posterior aspect of the left middle temporal gyrus (MTG), left fusiform gyrus (FFG), and left inferior temporal gyrus (ITG) (34). In addition, the acoustic signal of speech and the expressive language could be influenced by mild cognitive impairment as well. For example, magnetic resonance imaging (MRI) measures of the volume of the segmented gray matter of the frontal lobe were associated with acoustic measurements, with the result that an acoustic and MRI-based model can sufficiently predict the diagnosis of mild cognitive impairment (35). Although mild cognitive impairment was not investigated in the present study, these findings highlight a potential reason that comparable acoustic speech changes as described in this meta-analysis may also be present in earlier stages of Alzheimer’s disease. Further structural MRI scans of Alzheimer’s disease and mild cognitive impairment patients revealed that the left inferior parietal lobe (IPL), the right ITG, and the right FFG all contributed to lowering the pitch level (36). There was also a significant relationship between the right FFG, the left FFG and the loudness level, with the consequence that a reduction in the volume of both regions leads to a decreased loudness level (36). Finally, significant relationships between the right IPL and left FFG and speech rate were also investigated, with atrophy of these brain regions leading to a decrease in speech rate (36).

From the included acoustic parameters in this meta-analysis, rate-related parameters and the interruption-related parameters had the highest potential in the acoustic detection of AD, with an overall significant difference confirmed between healthy controls (p ≤ 0.01). Temporal-related measures were only evident in two of four acoustic parameters for significant detection of AD: mean speech segment duration and nPVI (p < 0.01). No significant effects were assessed by the total duration and mean pause duration between AD and HC, and can be excluded as potential acoustic detection parameters for AD.

The publications included generally exhibited a low risk of bias, with eight out of the eleven studies indicating a low risk. Heterogeneity varied from none to high, with three out of seven analyses showing no heterogeneity. Only one parameter demonstrated moderate heterogeneity, mean speech segment duration of temporal-related category. Mean pause duration and total duration of temporal-related, and speech rate of rate-related categories presented a high heterogeneity. On the one hand, this heterogeneity between studies underscores the complexity of speech dynamics in AD and requires a nuanced understanding of how different acoustic parameters reflect underlying neurological changes. On the other hand, duration measurements can vary greatly depending on how the author groups in the studies defined the minimum length of «speech segments» and «silent pauses». For example, the minimum length of silent pauses was set at 150 milliseconds (ms) (29) or even 100 ms (27), which is mostly longer than the onset time of voiceless stop consonants in English. Depending on the definition of silent pauses, the duration and number of mean speech segments may also vary and might explain moderate to large heterogeneity in these measurements.

This meta-analysis confirmed that individuals with AD revealed significant lower results in the performance of the two rate-related parameters of speech rate and articulation rate. Furthermore, the significance of these findings lies in their potential to inform non-invasive and cost-effective screening processes for AD, offering an alternative to the more traditional and often expensive diagnostic methods. Speech rate refers to the speed at which an individual speaks, measured in words per minute (37). Furthermore, speech rate also includes the presence of pauses (38). Another measurement of speech rate is the articulation rate which describe the rate at which someone can articulate each phoneme in a word with precision and clarity, without taking into account any pauses (39). In individuals with AD, the degeneration of brain cells can lead to a decline in the co-ordination and regulation of the muscles movements resulting in apraxia, which can be evident in speech and orofacial muscles (40). This apraxia further complicates the speech production process, making acoustic analysis an effective tool in capturing these subtle yet clinically relevant changes. This can cause a slowdown in speech as individuals struggle to express their thoughts (23, 24, 27). Additionally, the precision of articulation may decrease as individuals find it challenging to coordinate the movements of their lips, tongue, and jaw to produce each sound in a word (23, 24, 27, 32). Rate-related parameters are both closely related to temporal-related parameters as patients who have lower speech rate produce a shorter speech segment and vice versa (41). Therefore, the results from rate-related parameters are compatible with temporal-related parameters.

Also the mean speech segment duration revealed significant lower results in the AD groups as the healthy controls. This parameter refers to the average length of uninterrupted speech segments. The reduction in mean speech segment duration among individuals with AD could reflect difficulties in sustaining speech flow, possibly due to impaired cognitive planning and execution of language. The significant difference in mean speech segment duration between the two groups suggests that individuals with AD may exhibit shorter speech timing compared to control group (27, 31, 32). In individuals with AD, this specific aspect might be affected due to difficulties in maintaining coherent and fluent speech. This impairment in fluency suggests a disruption in the lexical-semantic network, critical for the organized retrieval of conceptual knowledge necessary for coherent speech production. This decline has been well correlated with a decrease in metabolic activity within certain regions of the brain, specifically the IFG located in the frontal lobe, as well as various regions within the temporal lobe (34, 42).

In two other parameters patients with AD performed significant higher results as the HC (voice breaks and nPVI). The nPVI which is based upon the temporal and durational aspects of voice is a measure of the normalized sequential variability index of syllabic intervals (25). It is a robust measurement for the purpose of quantifying the level of variability present in speech rhythm, one key element of prosody of speech (43). According to research findings, stress-timed languages such as English and German exhibit more significant variations in duration between consecutive vocalic intervals (section of speech between vowel onset and offset). This is attributed to the presence of both complex and simple syllabic structures in these languages. As a result, stress-timed languages tend to have higher nPVI values compared to syllable-timed languages like Spanish and French. Conversely, syllable-timed languages are anticipated to demonstrate less deviation in vocalic interval duration and consequently lower nPVI values (44). Based on the present meta-analysis, the rhythm of speech can be negatively affected by AD, resulting in impaired timing and coordination. Consequently, individuals with AD may exhibit increased variability in their speech rhythm, as indicated by higher values of nPVI (25, 26). Voice breaks, also known as one of the symptoms of voice disorders, are characterized by abrupt alterations in the sound of the voice, for example the melodic pitch curve, in which characteristic noises such as bubbling or tremor in the voice also occur (23). According to outcomes, the significant difference in voice breaks between the two groups suggests that individuals with AD may experience more frequent instabilities in pitch compared to HC individuals (23, 28). The increased incident of voice breaks in AD patients may suggest the presence of underlying physiological alterations in the vocal folds or the neural pathways implicated in vocal production process. The increased variability in speech rhythm and the presence of voice breaks in individuals with AD could potentially serve as acoustic markers of disease severity and progression, offering insights into the impact of AD on motor control and speech planning.

The present results demonstrated opportunities of the disease management to use acoustic measures for the detection of AD but also to monitor the progression, stability, or process of the disease. Furthermore, these results not only highlight the potential of acoustic measures for the detection of AD but also underscore the importance of further refining these parameters to enhance their diagnostic utility.

The limitations of this meta-analysis not only concern the relevance of the results, but also offer valuable perspectives for future research. First, the inclusion of studies published only in written English limits the diversity and scope of the data analyzed, potentially overlooking valuable research in other published languages. Second, each parameter was only evaluated in four studies at top, and this issue highlights the paucity of research in this area. Third, the bias of risk was observed in three studies as moderate. Another drawback relates to comparability and reproducibility between the used software in the studies, and equally important, some of the papers used software that was not known (see Table 1). Fourth, although acoustic rate measures were promising in the present meta-analysis between AD and HC, a slowed speech rate can also occur in other neurological diseases based on motor impairment and needs to be further investigated with regard to a differential diagnosis in neurodegenerative diseases (e.g., Parkinson’s or Amyotrophic Lateral Sclerosis). Fifth, some studies used a small number of sample sizes such as Gonzalez-Moreira et al. (24) and Bose et al. (30). Sixth, two parameters (i.e., nPVI and voice breaks) included in this meta-analysis were analyzed by the same groups of authors but in different publications. In such situations, it becomes more challenging to determine whether the observed effects and low heterogeneity (e.g., potential overlap of participants between the included studies) are truly generalizable across all cohorts, languages, and so on. More comprehensive research is necessary to confirm the present results of these two parameters. Seventh, the most included studies relied on clinical diagnoses of Alzheimer’s disease (AD) and did not provide biomarker confirmation of the underlying AD neuropathology. As a result, the findings of this meta-analysis should be interpreted as demonstrating a distinction between individuals with and without dementia, rather than specifically between those with and without biological AD. This distinction is important, as it highlights the potential limitations of using purely clinical diagnostic criteria in research studies. Despite these limitations, the current meta-analysis provides valuable insights into the potential utility of acoustic markers in distinguishing between individuals with and without dementia. Future studies that incorporate biomarker data using more stringent diagnostic criteria will be important to further refine our understanding of the relationship between acoustic markers and AD neuropathology. Eighth, the present meta-analysis used two different types of continuous speech tasks (reading and spontaneous). There might be evidence that there are significant differences in the outcomes of acoustic parameters between these two speech tasks, for example, pauses might be fewer in reading than in continuous speech (45). However, there also other studies which found no significant differences in acoustic speech parameters (46, 47). Thus, we would like to emphasize that a possible influence of the two different speech tasks in the same category of continuous speech cannot be excluded and should be taken into account in future studies. Ninth, just a few of acoustic parameters have been investigated in these studies, but there are much more which could be of interest such as the cepstral analysis. This analysis is being vastly utilized in the assessment and treatment of patients with voice disorders, motor-speech disorder, other neurodegenerative diseases, and hypokinetic disorders (48–53). Furthermore, the exploration of additional acoustic parameters such as cepstral analysis could enrich our understanding of speech alterations in AD, paving the way for more sophisticated diagnostic models.

## Conclusion

The analysis of the gathered evidence through meta-analysis illuminated the susceptibility of specific speech signal components to alteration in the context of AD. These findings not only contribute to our understanding of the acoustic manifestations of AD but also highlight the broader implications for early detection and monitoring of the disease. Individuals with AD, as opposed to those who have normal cognitive function, tend to have shorter utterances, speak at a slower pace, and show more variability in voice breaks, which impacts their speech rhythm. Moreover, the potential integration of acoustic analysis into routine diagnostic and monitoring practices represents a significant advancement in our approach to managing AD, emphasizing the need for interdisciplinary collaboration to harness the full potential of this technology. As a result, these discoveries, in conjunction with other accessible resources, can aid healthcare professionals in attaining a more accurate diagnosis of AD in a manner that is relatively economical and straightforward to implement.

## Electronic Supplementary Material

Supplementary material, approximately 31 KB.
