# Supplementary material for: Acoustic Speech Analysis in Alzheimer’s Disease: A Systematic Review and Meta-Analysis
Source: J Prev Alzheimers Dis. 2024 Aug 13;11(6):1789–97. doi: 10.14283/jpad.2024.132 (PMC11573841; doi:10.14283/jpad.2024.132)
Supplement: Supplementary file 1 — Supplementary material, approximately 31 KB. [file 42414_2024_132_MOESM1_ESM.docx]

**Appendices**

**Appendix 1:** Search strategies for systematic review

| **Database** | **Search strategy** |
| --- | --- |
| MEDLINE | ("voice" [All Fields] OR "acoustics" [All Fields] OR "acoustic features" [All Fields] OR "acoustic measurements" [All Fields] OR "acoustic measures" [All Fields] OR "acoustic analysis" [All Fields] OR "acoustic analyses" [All Fields] OR "speech analysis" [All Fields] OR "speech features" [All Fields] AND ("Alzheimer" [All Fields] OR "Alzheimer’s disease" [All Fields] OR "dementia" [All Fields]) |
| CENTRAL | acoustic AND (analysis OR measure OR measure OR measurements OR features) AND (Alzheimer OR dementia) |
| Scopus | "acoustic features" OR "acoustic measurements" OR "acoustic measures" OR "speech features" OR "speech analysis" OR "acoustic analysis" OR "acoustic analyses" AND "Alzheimer" OR "Alzheimer’s disease" OR "dementia". |
| Web of Science | acoustic AND (analysis OR measure OR measure OR measurements OR features) AND (Alzheimer OR dementia). |

**Appendix 2:** Risk of Bias analyzed with JBI Critical Appraisal Checklist for Analytical Cross-sectional Studies

| **Meilán et al. (2014)** | | | | | |
| --- | --- | --- | --- | --- | --- |
| **Not applicable** | **Unclear** | **No** | **Yes** | **Item** | **Number** |
|  |  |  | * | Were the criteria for inclusion in the sample clearly defined? | 1 |
|  | * |  |  | Were the study subjects and the setting described in detail? | 2 |
|  |  |  | * | Was the exposure measured in a valid and reliable way? | 3 |
|  |  |  | * | Were objective, standard criteria used for measurement of the condition? | 4 |
|  |  |  | * | Were confounding factors identified? | 5 |
|  |  |  | * | Were strategies to deal with confounding factors stated? | 6 |
|  |  |  | * | Were the outcomes measured in a valid and reliable way? | 7 |
|  |  |  | * | Was appropriate statistical analysis used? | 8 |

| **Gonzalez-Moreira et al. (2015)** | | | | | |
| --- | --- | --- | --- | --- | --- |
| **Not applicable** | **Unclear** | **No** | **Yes** | **Item** | **Number** |
|  |  |  | * | Were the criteria for inclusion in the sample clearly defined? | 1 |
|  | * |  |  | Were the study subjects and the setting described in detail? | 2 |
|  |  |  | * | Was the exposure measured in a valid and reliable way? | 3 |
|  |  |  | * | Were objective, standard criteria used for measurement of the condition? | 4 |
|  | * |  |  | Were confounding factors identified? | 5 |
|  | * |  |  | Were strategies to deal with confounding factors stated? | 6 |
|  |  |  | * | Were the outcomes measured in a valid and reliable way? | 7 |
|  |  |  | * | Was appropriate statistical analysis used? | 8 |

| **Martinez-Sanchez et al. (2017)** | | | | | |
| --- | --- | --- | --- | --- | --- |
| **Not applicable** | **Unclear** | **No** | **Yes** | **Item** | **Number** |
|  |  |  | * | Were the criteria for inclusion in the sample clearly defined? | 1 |
|  | * |  |  | Were the study subjects and the setting described in detail? | 2 |
|  |  |  | * | Was the exposure measured in a valid and reliable way? | 3 |
|  |  |  | * | Were objective, standard criteria used for measurement of the condition? | 4 |
|  |  |  | * | Were confounding factors identified? | 5 |
|  |  |  | * | Were strategies to deal with confounding factors stated? | 6 |
|  |  |  | * | Were the outcomes measured in a valid and reliable way? | 7 |
|  |  |  | * | Was appropriate statistical analysis used? | 8 |

| **Meilán el al. (2018)** | | | | | |
| --- | --- | --- | --- | --- | --- |
| **Not applicable** | **Unclear** | **No** | **Yes** | **Item** | **Number** |
|  |  |  | * | Were the criteria for inclusion in the sample clearly defined? | 1 |
|  |  |  | * | Were the study subjects and the setting described in detail? | 2 |
|  |  |  | * | Was the exposure measured in a valid and reliable way? | 3 |
|  |  |  | * | Were objective, standard criteria used for measurement of the condition? | 4 |
|  |  |  | * | Were confounding factors identified? | 5 |
|  |  |  | * | Were strategies to deal with confounding factors stated? | 6 |
|  |  |  | * | Were the outcomes measured in a valid and reliable way? | 7 |
|  |  |  | * | Was appropriate statistical analysis used? | 8 |

| **De Loozea et al. (2018)** | | | | | |
| --- | --- | --- | --- | --- | --- |
| **Not applicable** | **Unclear** | **No** | **Yes** | **Item** | **Number** |
|  |  |  | * | Were the criteria for inclusion in the sample clearly defined? | 1 |
|  |  |  | * | Were the study subjects and the setting described in detail? | 2 |
|  |  |  | * | Was the exposure measured in a valid and reliable way? | 3 |
|  |  |  | * | Were objective, standard criteria used for measurement of the condition? | 4 |
|  |  |  | * | Were confounding factors identified? | 5 |
|  |  |  | * | Were strategies to deal with confounding factors stated? | 6 |
|  |  |  | * | Were the outcomes measured in a valid and reliable way? | 7 |
|  |  |  | * | Was appropriate statistical analysis used? | 8 |

| **Qiao et al. (2020)** | | | | | |
| --- | --- | --- | --- | --- | --- |
| **Not applicable** | **Unclear** | **No** | **Yes** | **Item** | **Number** |
|  |  |  | * | Were the criteria for inclusion in the sample clearly defined? | 1 |
|  |  |  | * | Were the study subjects and the setting described in detail? | 2 |
|  |  |  | * | Was the exposure measured in a valid and reliable way? | 3 |
|  |  |  | * | Were objective, standard criteria used for measurement of the condition? | 4 |
|  |  |  | * | Were confounding factors identified? | 5 |
|  |  |  | * | Were strategies to deal with confounding factors stated? | 6 |
|  |  |  | * | Were the outcomes measured in a valid and reliable way? | 7 |
|  |  |  | * | Was appropriate statistical analysis used? | 8 |

| **Frankenberg et al. (2021)** | | | | | |
| --- | --- | --- | --- | --- | --- |
| **Not applicable** | **Unclear** | **No** | **Yes** | **Item** | **Number** |
|  | * |  |  | Were the criteria for inclusion in the sample clearly defined? | 1 |
|  | * |  |  | Were the study subjects and the setting described in detail? | 2 |
|  | * |  |  | Was the exposure measured in a valid and reliable way? | 3 |
|  |  |  | * | Were objective, standard criteria used for measurement of the condition? | 4 |
|  |  |  | * | Were confounding factors identified? | 5 |
|  |  |  | * | Were strategies to deal with confounding factors stated? | 6 |
|  |  |  | * | Were the outcomes measured in a valid and reliable way? | 7 |
|  |  |  | * | Was appropriate statistical analysis used? | 8 |

| **Bose et al. (2021)** | | | | | |
| --- | --- | --- | --- | --- | --- |
| **Not applicable** | **Unclear** | **No** | **Yes** | **Item** | **Number** |
|  | * |  |  | Were the criteria for inclusion in the sample clearly defined? | 1 |
|  | * |  |  | Were the study subjects and the setting described in detail? | 2 |
|  |  |  | * | Was the exposure measured in a valid and reliable way? | 3 |
|  |  |  | * | Were objective, standard criteria used for measurement of the condition? | 4 |
|  |  |  | * | Were confounding factors identified? | 5 |
|  |  |  | * | Were strategies to deal with confounding factors stated? | 6 |
|  |  |  | * | Were the outcomes measured in a valid and reliable way? | 7 |
|  |  |  | * | Was appropriate statistical analysis used? | 8 |

| **Martinez-Sanchez et al. (2018)** | | | | | |
| --- | --- | --- | --- | --- | --- |
| **Not applicable** | **Unclear** | **No** | **Yes** | **Item** | **Number** |
|  |  |  | * | Were the criteria for inclusion in the sample clearly defined? | 1 |
|  | * |  |  | Were the study subjects and the setting described in detail? | 2 |
|  |  |  | * | Was the exposure measured in a valid and reliable way? | 3 |
|  |  |  | * | Were objective, standard criteria used for measurement of the condition? | 4 |
|  | * |  |  | Were confounding factors identified? | 5 |
|  | * |  |  | Were strategies to deal with confounding factors stated? | 6 |
|  |  |  | * | Were the outcomes measured in a valid and reliable way? | 7 |
|  |  |  | * | Was appropriate statistical analysis used? | 8 |

| **Cho et al. (2022)** | | | | | |
| --- | --- | --- | --- | --- | --- |
| **Not applicable** | **Unclear** | **No** | **Yes** | **Item** | **Number** |
|  |  |  | * | Were the criteria for inclusion in the sample clearly defined? | 1 |
|  |  |  | * | Were the study subjects and the setting described in detail? | 2 |
|  |  |  | * | Was the exposure measured in a valid and reliable way? | 3 |
|  |  |  | * | Were objective, standard criteria used for measurement of the condition? | 4 |
|  |  |  | * | Were confounding factors identified? | 5 |
|  |  |  | * | Were strategies to deal with confounding factors stated? | 6 |
|  |  |  | * | Were the outcomes measured in a valid and reliable way? | 7 |
|  |  |  | * | Was appropriate statistical analysis used? | 8 |

| **Yamada et al. (2023)** | | | | | |
| --- | --- | --- | --- | --- | --- |
| **Not applicable** | **Unclear** | **No** | **Yes** | **Item** | **Number** |
|  |  |  | * | Were the criteria for inclusion in the sample clearly defined? | 1 |
|  | * |  |  | Were the study subjects and the setting described in detail? | 2 |
|  |  |  | * | Was the exposure measured in a valid and reliable way? | 3 |
|  |  |  | * | Were objective, standard criteria used for measurement of the condition? | 4 |
|  |  |  | * | Were confounding factors identified? | 5 |
|  |  |  | * | Were strategies to deal with confounding factors stated? | 6 |
|  |  |  | * | Were the outcomes measured in a valid and reliable way? | 7 |
|  |  |  | * | Was appropriate statistical analysis used? | 8 |
